# Leser-Trélat Sign as a Marker for Underlying Pancreatic Cancer

**DOI:** 10.5811/cpcem.1248

**Published:** 2023-08-09

**Authors:** Kalpit Modi, Richard Chen, Layla Abubshait

**Affiliations:** Einstein Healthcare Network, Einstein Medical Center Montgomery Emergency Department, East Norriton, Pennsylvania

**Keywords:** Leser-Trélat, cancer, malignancy, seborrheic keratosis

## Abstract

**Case Presentation:**

Early diagnosis and rapid treatment of cancer is essential for good clinical outcomes for patients. In this case, an 85-year-old man presented with failure to thrive and was noted to have rapid-onset, multiple seborrheic keratoses (Leser-Trélat sign) on his chest and back. He was ultimately diagnosed with pancreatic cancer using computed tomography.

**Discussion:**

Leser-Trélat sign is a rare cutaneous marker for underlying malignancy. Identification of this sign can help guide diagnostic imaging and lab work to identify an occult internal malignancy, resulting in more rapid diagnosis, earlier treatment, and potentially better clinical outcomes.

## CASE PRESENTATION

**Image 1:**
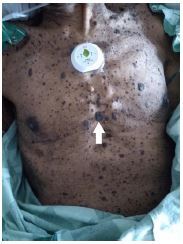
Multiple seborrheic keratoses (arrow) on the chest and abdomen.

An 85-year-old man with a history of chronic obstructive pulmonary disease, chronic kidney disease, congestive heart failure, and atrial fibrillation presented to the emergency department (ED) via ambulance for failure to thrive. The patient’s home-care nurse was concerned due to his worsening weakness, development of peripheral edema, decreasing appetite, and general deterioration over the prior week. The patient had no complaints, aside from diarrhea, and was not sure why his home-care nurse had called for an ambulance. On arrival to the ED, the patient was hemodynamically stable and oxygenating well on a baseline of two liters of oxygen via nasal cannula. He appeared thin and frail and had significant generalized weakness when moving his extremities. Cutaneous exam showed numerous seborrheic keratoses on his chest, abdomen, and back (Image 1).

**Image 2:**
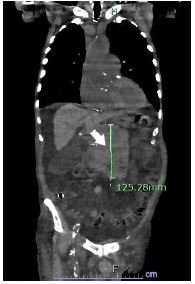
Pancreatic mass (arrow) diagnosed via computed tomography of the abdomen and pelvis in coronal view.

**Image 3:**
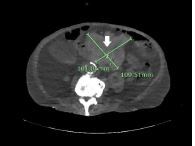
Pancreatic mass (arrow) diagnosed via computed tomography of the abdomen and pelvis in axial view.

The patient had no abdominal tenderness to palpation. However, he did have significant lower extremity edema. His constellation of symptoms in combination with the cutaneous finding (absent on skin exams from his admission two months prior) raised suspicion that his eruptive skin lesions were a manifestation of the Leser-Trélat sign (LTS). He underwent computed tomography (CT) of the abdomen and pelvis, which revealed a lobular mass in the pancreatic body measuring 10 centimeters (cm) × 11 cm × 12 cm, concerning for likely malignancy (Images 2, 3). After admission to the hospital, the patient and family made the joint decision to transition him to hospice care, opting to forego further medical management of the underlying cancer.

## DISCUSSION

Leser-Trélat sign is a rare cutaneous marker for underlying malignancy.^1^,^2^ It manifests with a sudden appearance or rapid accumulation of multiple seborrheic keratoses on the chest, abdomen, or back.^2^ Seborrheic keratoses are waxy-textured papules that are black or brown in color and characterized by their appearance of being stuck onto the skin.^3^ Seborrheic keratoses are themselves benign skin growths that grow slowly over a number of years, but their accumulation or sudden appearance (often within one year) can be a sign of an underlying malignancy.^1^,^4^ The most common malignancies associated with LTS are those of the gastrointestinal tract, in particular, gastric adenocarcinoma.^1^ The pathogenesis of LTS is still unknown but hypothesized to be paraneoplastic in nature.^1^,^2^,^4^

**CPC-EM Capsule**

What do we already know about this clinical entity?

*The Leser-Trélat sign, the rapid appearance or accumulation of seborrheic keratoses, is a rare cutaneous marker that can point to an underlying malignancy.*

What is the major impact of the image(s)?

*The images show a manifestation of the Leser-Trélat sign in a patient ultimately diagnosed with pancreatic cancer.*

How might this improve emergency medicine practice?

*Identification of the Leser-Trélat sign in the emergency department can lead to earlier diagnosis and treatment of the underlying cancer.*

In the ED, incidental cutaneous findings are often disregarded in favor of more pressing pathologies; however, identification of LTS can direct imaging that could lead to earlier diagnosis and treatment of the underlying cancer.^5^ Prognosis of patients with LTS is often poor since the cancer is usually in a more advanced stage.^2^,^5^ To clarify, it is not always necessary to search for an underlying malignancy in the ED for patients with LTS. Instead, identification of LTS is important because it spurs physicians to arrange close follow-up with an outpatient dermatologist for further testing.
